# Novel β-catenin target genes identified in thalamic neurons encode modulators of neuronal excitability

**DOI:** 10.1186/1471-2164-13-635

**Published:** 2012-11-17

**Authors:** Marta B Wisniewska, Andrzej Nagalski, Michal Dabrowski, Katarzyna Misztal, Jacek Kuznicki

**Affiliations:** 1 International Institute of Molecular and Cell Biology, Laboratory of Neurodegeneration, ul. Ks, Trojdena 4, 02-109, Warsaw, Poland; 2 Nencki Institute of Experimental Biology, Laboratory of Transcription Regulation, ul, Pasteura 3, 02-093, Warsaw, Poland; 3 Nencki Institute of Experimental Biology, Laboratory of Calcium Binding Proteins, ul, Pasteura 3, 02-093, Warsaw, Poland

**Keywords:** β-catenin, Wnt, LEF1/TCF, Transcription regulation, Adult brain, Neurons, Thalamus

## Abstract

**Background:**

LEF1/TCF transcription factors and their activator β-catenin are effectors of the canonical Wnt pathway. Although Wnt/β-catenin signaling has been implicated in neurodegenerative and psychiatric disorders, its possible role in the adult brain remains enigmatic. To address this issue, we sought to identify the genetic program activated by β-catenin in neurons. We recently showed that β-catenin accumulates specifically in thalamic neurons where it activates *Cacna1g* gene expression. In the present study, we combined bioinformatics and experimental approaches to find new β-catenin targets in the adult thalamus.

**Results:**

We first selected the genes with at least two conserved LEF/TCF motifs within the regulatory elements. The resulting list of 428 putative LEF1/TCF targets was significantly enriched in known Wnt targets, validating our approach. Functional annotation of the presumed targets also revealed a group of 41 genes, heretofore not associated with Wnt pathway activity, that encode proteins involved in neuronal signal transmission. Using custom polymerase chain reaction arrays, we profiled the expression of these genes in the rat forebrain. We found that nine of the analyzed genes were highly expressed in the thalamus compared with the cortex and hippocampus. Removal of nuclear β-catenin from thalamic neurons *in vitro* by introducing its negative regulator Axin2 reduced the expression of six of the nine genes. Immunoprecipitation of chromatin from the brain tissues confirmed the interaction between β-catenin and some of the predicted LEF1/TCF motifs. The results of these experiments validated four genes as authentic and direct targets of β-catenin: *Gabra3* for the receptor of GABA neurotransmitter, *Calb2* for the Ca^2+^-binding protein calretinin, and the *Cacna1g* and *Kcna6* genes for voltage-gated ion channels. Two other genes from the latter cluster, *Cacna2d2* and *Kcnh8*, appeared to be regulated by β-catenin, although the binding of β-catenin to the regulatory sequences of these genes could not be confirmed.

**Conclusions:**

In the thalamus, β-catenin regulates the expression of a novel group of genes that encode proteins involved in neuronal excitation. This implies that the transcriptional activity of β-catenin is necessary for the proper excitability of thalamic neurons, may influence activity in the thalamocortical circuit, and may contribute to thalamic pathologies.

## Background

β-catenin is an armadillo family protein that serves as a gene expression regulator in canonical Wnt signaling, in addition to its function in cell adhesion [1,2]. The canonical Wnt pathway involves the inhibition of GSK3α/β (i.e., a negative regulator of β-catenin), dissociation of the β-catenin destruction complex that contains APC and Axin proteins, and subsequent accumulation of the cytoplasmic pool of β-catenin that can translocate into the nucleus and activate LEF1/TCF transcription factors [3,4]. The crucial role of this pathway in cell differentiation and proliferation is well established, and mutations in its components lead to severe malformations in developing embryos and cancer in adults [5].

The nuclear mediators of canonical Wnt signaling include the transcription factors LEF1, TCF7 (also known as TCF1), TCF7L1 (also known as TCF3), and TCF7L2 (also known as TCF4), which are members of the high mobility group (HMG) family [6-8]. The HMG DNA binding domain of LEF1/TCFs recognizes the WWCAAAG consensus sequence. The *N*-terminus of LEF1/TCF recruits β-catenin, which does not bind to DNA itself but has a strong transactivation domain. The remaining regulatory domains and alternatively spliced *C*-termini of the proteins vary between these members and provide a molecular basis for the diversity and context dependence of LEF1/TCFs function. *Lef1/Tcf* genes are highly expressed during mouse embryogenesis, but their transcripts are virtually undetectable postnatally [9,10], except for intestinal and hair follicle stem cells [11,12]. Surprisingly, high levels of *Lef1* and *Tcf7l2* expression have been observed in the thalamus in the adult brain [13-16].

Growing evidence suggests the involvement of canonical Wnt signaling in the proper functioning of the adult central nervous system [17]. Aberrant regulation of this pathway has been associated with psychotic and affective disorders (e.g., major depression, bipolar disorder, and schizophrenia) [18-24] and neurodegenerative diseases [25-29]. However, the physiological role of Wnt/β-catenin in the adult brain is far from understood. The transcriptional activity of β-catenin has been implicated in adult neurogenesis [30-33] and gliogenesis [34]. These functions resemble the role of Wnt/β-catenin signaling during neuronal development. Nonetheless, nuclear β-catenin has also been shown to accumulate in mature neurons - in hippocampal cells upon NMDA (N-Methyl-D-aspartate) receptor activation [35-37] and constitutively in thalamic cells [38]. The identification of β-catenin target genes in neurons may provide insights into its role in these cells and the adult brain.

Most of the approximately 100 known β-catenin targets are involved in development and cancer. In differentiated neurons, only a few genes have been shown to be activated by β-catenin; several are already known Wnt target genes that are not neuron-specific [35-37]. As we recently demonstrated, a new target, *Cacna1G*, encodes voltage-gated Ca^2+^ channels [16]. In this work, we identified *in silico* and experimentally validated a novel group of β-catenin-LEF1/TCF targets in thalamic neurons, consisting of genes that encode proteins that are important for neuronal function, including voltage- and ligand-gated ion channels and the Ca^2+^-binding protein calretinin. This indicates a role for β-catenin and LEF1/TCF transcription factors in the maintenance of neuronal excitability.

## Results

### Putative LEF1/TCF target genes identified in silico are enriched in known Wnt targets

**Figure 1:**
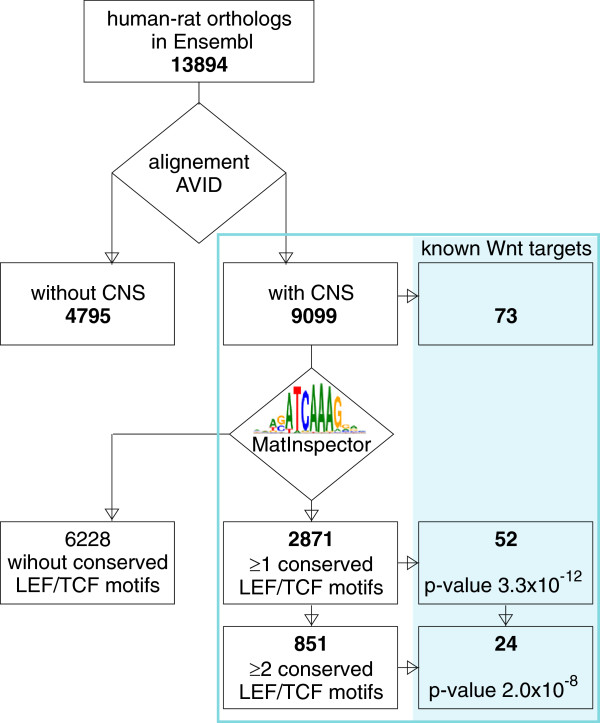
**Bioinformatics identification of putative LEF1/TCF target genes.** The diagram presents the successive steps to select putative LEF1/TCF targets, beginning from the group of human-rat orthologs in the Ensembl database. Groups of genes are in rectangles, and computational procedures are in diamonds. In the blue frame, the crossing of our *in silico*-selected genes with known Wnt/β-catenin targets is shown. The *p* values (Fisher’s Exact test) confirmed the enrichment of genes with at least one and at least two LEF1/TCF binding sites (predicted with Matinspector using Genomatix family V$LEFF) with the known targets.

To find putative β-catenin target genes, we screened for LEF1/TCF family motifs in rat and human conserved regions within 10 kb upstream and downstream of transcription start sites (TSSs). We relied on the fact that enhancers are enriched in proximity to TSSs [39], and the clustering of TCF7L2-bound regions occurs within 10 kb of TSSs in colorectal cancer [40]. Using established bioinformatics tools with their default parameters, we identified 2,871 genes with at least one rat-to-human conserved LEF1/TCF motif and 851 genes that contain at least two LEF1/TCF motifs in the same conserved noncoding sequences (CNSs). These two lists were compared with experimentally established Wnt target genes cataloged at http://www.stanford.edu/group/nusselab/cgi-bin/wnt/target_genes (accessed January 11, 2012; Figure 1). The degree of overlap between our lists and the list on the Wnt homepage shows that the Wnt targets are highly overrepresented among the genes that contain the predicted conserved LEF1/TCF binding site. This positively validated our bioinformatics approach to identifying LEF1/TCF targets. For further analysis, we selected only the genes with at least two LEF1/TCF motifs in one regulatory element, expecting fewer false-positive hits in this group. This presumption was based on the observation that more than 30% of the genes that contain pairs of TCF7L2 motifs in the same CNS display Wnt target-like patterns during mouse development [41]. We excluded genes with a “NULL” or “PREDICTED” description in the Ensembl database, creating a final list of 428 rat genes (Additional file 1), which we considered putative LEF1/TCF targets.

### Putative LEF1/TCF targets are enriched in neuron-specific genes (GO analysis)

We performed a gene ontology (GO) analysis of the 428 putative targets using DAVID Functional Annotation tools (http://david.abcc.ncifcrf.gov; accessed January 11, 2012) to identify functionally related groups of genes. As expected, our list was highly enriched in genes involved in development, patterning, and cell proliferation (Table 1). Other predictable categories of molecular function and biological processes were overrepresented: specific DNA binding (transcription factors), Wnt signaling, and cell migration, adhesion, and death. Unexpectedly, we found statistically significant enrichment in genes implicated in neuronal function, including genes for synaptic proteins and voltage-gated cation channels (VGCCs). Based on this analysis, we hypothesized that β-catenin-LEF1/TCF can regulate specific neuronal genetic programs. We collected the genes that were annotated with “synapse” and “gated-channel activity” terms and included a few other genes that failed to be annotated with the above GO terms, although they obviously belong to these categories (e.g., the γ-aminobutyric acid [GABA] receptor-associated protein gene *Gabarap*). Several genes were also excluded because they were not brain-specific (e.g., *Chrnd*, which encodes a neuromuscular cholinergic receptor). The final collection contained 41 putative neuronal LEF1/TCF targets grouped into five categories: nine VGCCs, 11 neurotransmitter receptors, eight proteins involved in synaptic vesicle organization, 10 synaptic structural proteins, and three other proteins implicated in synaptic transmission (Table 2). All of these gene targets were chosen for experimental validation.

**Table 1:** Overrepresented GO terms in the predicted LEF1/TCF target genes

***GO term***	***Number of genes***	***p value***
**Three top terms**		
Neuron differentiation	67	1.9E-27
Pattern specification	45	3.4E-21
Sequence-specific DNA binding	60	4.7E-21
**Selection of other expected terms**		
Regulation of cell development	35	1.7E-14
Cell migration	33	8.0E-12
Cell proliferation	26	1.75E-7
Cell adhesion	35	1.4E-6
Regulation of cell death	43	9.6E-6
Wnt receptor signaling pathway	11	2.0E-4
**Selection of novel terms related to neuronal function**		
Synapse	32	3.6E-8
Gated-channel activity	20	2.8E-4

The *p* values are based on DAVID Bioinformatics Resources (see Materials and Methods).

**Table 2:** Putative LEF1/TCF target genes that encode proteins with specific neuronal functions

***No.***	***Gene symbol (RGD)***	***Protein name; description***
**Voltage-gated cation channels**		
1	*Cacna1g*	Ca^2+^ channel, voltage-gated, α1G subunit
2	*Cacna2d1*	Ca^2+^ channel, voltage-gated, α2/δ1 subunit
3	*Cacna2d2*	Ca^2+^ channel, voltage-gated, α2/δ2 subunit
4	*Kcna1*	K^+^ voltage-gated, shaker-related subfamily member 1
5	*Kcna6*	K^+^ voltage-gated, shaker-related subfamily member 6
6	*Kcnh4*	K^+^ voltage-gated, subfamily H member 4
7	*Kcnh8*	K^+^ voltage-gated, subfamily H member 8
8	*Kcnj3*	K^+^ inwardly rectifying channel, subfamily J member 3
9	*Scn8a*	Na^+^ voltage-gated, type VIII, alpha subunit
**Neurotransmitter receptors**		
10	*Cnr1*	Cannabinoid CB_1_ receptor; metabotropic
11	*Drd3*	Dopamine D_3_ receptor; metabotropic
12	*Gabra3*	GABA_Aα3_ receptor; ionotropic (Cl^-^)
13	*Gabrg2*	GABA_Aγ2_ receptor; ionotropic (Cl^-^)
14	*Glra1*	Glycine α_1_ receptor; ionotropic
15	*Gria3*	Glutamate AMPA 3 receptor; ionotropic
16	*Grid2*	Glutamate δ_2_ receptor; ionotropic
17	*Grik3*	Glutamate kainate 3 receptor; ionotropic
18	*Htr1b*	5-hydroxytryptamine (serotonin) 1B receptor; metabotropic
19	*Htr3a*	Serotonin 3A receptor; ionotropic
20	*Htr3b*	Serotonin 3B receptor; ionotropic
**Synaptic vesicle proteins**		
21	*Syt1*	Synaptotagmin I; membrane protein, Ca^2+^ sensor involved in vesicle trafficking and neurotransmitter release
22	*Syt4*	Synaptotagmin IV; membrane protein, vesicle trafficking and neurotransmitter release
23	*Syt5*	Synaptotagmin V; membrane protein, Ca^2+^ sensor involved in vesicle trafficking and neurotransmitter release
24	*Syt6*	Synaptotagmin VI; membrane protein, Ca^2+^ sensor involved in vesicle trafficking and neurotransmitter release
25	*Syt7*	Synaptotagmin VII; membrane protein, Ca^2+^ sensor involved in vesicle trafficking and neurotransmitter release
26	*Syp*	Synaptophysin; synaptic vesicle organization
27	*Vamp2*	Vesicle-associated membrane protein 2; vesicle transport and membrane fusion during exocytosis
28	*Vamp3*	Vesicle-associated membrane protein 3; vesicle transport and membrane fusion during exocytosis
**Structural synaptic proteins**		
29	*Cdh2*	Cadherin 2 (alias N-cadherin); hemophilic cell adhesion molecule in excitatory synapses
30	*Cnksr2*	Connector enhancer of kinase suppressor of Ras 2; scaffold protein, complex assembly of synaptic proteins
31	*Dfnb31*	Deafness autosomal recessive 31; PDZ scaffold protein, facilitates synaptic transmission
32	*Gabarap*	GABA_A_ receptor-associated protein; involved in clustering of neurotransmitter receptors
33	*Lin7a*	Homolog a of *C. elegans* lin-7; PDZ scaffold protein, localizes vesicles and receptors to the plasma membrane
34	*Map1b*	Microtubule-associated protein; involved in synaptic plasticity
35	*Mpdz*	Multiple PDZ domain protein; PDZ scaffold protein, component of NMDA receptor signaling complex
36	*Nlgn1*	Neuroligin 1; synaptic cell adhesion molecule, maintains synaptic junctions by binding β-neurexins
37	*Nlgn2*	Neuroligin 2; synaptic cell-adhesion molecule, maintains synaptic junctions by binding β-neurexins
38	*Nrxn3*	Neurexins 3; synaptic cell-adhesion molecule; maintains synaptic junctions by binding neuroligins
**Other proteins involved in modulation of signal transmission**		
39	*Gjd2*	Gap junction protein delta; member of the connexin family, in gap-junction channels of electrical synapses
40	*App*	Amyloid β (A4) precursor protein; cell surface receptor involved in synaptic plasticity
41	*Calb2*	Calbindin 2 (alias calretinin); Ca^2+^-binding protein, modulates neuronal excitability

The gene symbols are based on the Rat Genome Database (RGD).

### VGCC genes with conserved LEF1/TCF motifs compared with all other VGCC genes display preferential expression in the thalamus (RT-qPCR arrays)

To initially validate our bioinformatics predictions, we concentrated on the VGCC group. The relative mRNA levels of the ensemble of VGCC genes were measured in three different parts of the forebrain: thalamus, cortex, and hippocampus. We expected that the VGCC genes that are postulated LEF1/TCF targets would be preferentially expressed in the thalamus because nuclear β-catenin and LEF1 and TCF7L2 transcription factors are present in thalamic neurons but not cortical or hippocampal neurons [13-16]. The comparative expression analysis was performed using custom-designed quantitative real-time polymerase chain reaction (RT-qPCR) arrays with six independent preparations from each brain region. The arrays contained all VGCC genes that have CNSs (Table 3), excluding a few genes that are known to be expressed only in non-neuronal tissues.

**Table 3:** List of genes that encode voltage-gated cation channels included in the custom “VGCC” arrays

***No.***	***Gene symbol (RGD)***	***Channel type***	***Subunit type***	**Ensembl gene ID**
**1**	***Cacna1g***	**Ca**^**2+**^	**α1, pore**	**ENSRNOG00000002981**
2	*Cacna1h*	Ca^2+^	α1, pore	ENSRNOG00000033893
3	*Cacna1i*	Ca^2+^	α1, pore	ENSRNOG00000029914
4	*Cacnb1*	Ca^2+^	β, auxiliary	ENSRNOG00000004518
5	*Cacnb2*	Ca^2+^	β, auxiliary	ENSRNOG00000018378
6	*Cacnb3*	Ca^2+^	β, auxiliary	ENSRNOG00000012489
**7**	***Cacna2d1***	**Ca**^**2+**^	**α2δ, auxiliary**	**ENSRNOG00000033531**
**8**	***Cacna2d2***	**Ca**^**2+**^	**α2δ, auxiliary**	**ENSRNOG00000015835**
9	*Cacna2d3*	Ca^2+^	α2δ, auxiliary	ENSRNOG00000031287
**10**	***Kcna1***	**K**^**+**^	**α, pore**	**ENSRNOG00000019750**
11	*Kcna3*	K^+^	α, pore	ENSRNOG00000018116
12	*Kcna4*	K^+^	α, pore	ENSRNOG00000004918
**13**	***Kcna6***	**K**^**+**^	**α, pore**	**ENSRNOG00000019753**
14	*Kcnb1*	K^+^	α, pore	ENSRNOG00000008204
15	*Kcnb2*	K^+^	α, pore	ENSRNOG00000028991
16	*Kcnc1*	K^+^	α, pore	ENSRNOG00000011380
17	*Kcnc2*	K^+^	α, pore	ENSRNOG00000004077
18	*Kcnc3*	K^+^	α, pore	ENSRNOG00000019959
19	*Kcnd2*	K^+^	α, pore	ENSRNOG00000029610
20	*Kcnd3*	K^+^	α, pore	ENSRNOG00000014686
21	*Kcng3*	K^+^	α, pore	ENSRNOG00000004535
22	*Kcng4*	K^+^	α, pore	ENSRNOG00000015746
23	*Kcnh2*	K^+^	α, pore	ENSRNOG00000009872
24	*Kcnh3*	K^+^	α, pore	ENSRNOG00000032075
**25**	***Kcnh4***	**K**^**+**^	**α, pore**	**ENSRNOG00000018790**
26	*Kcnh5*	K^+^	α, pore	ENSRNOG00000009542
27	*Kcnh6*	K^+^	α, pore	ENSRNOG00000008078
28	*Kcnh7*	K^+^	α, pore	ENSRNOG00000007528
**29**	***Kcnh8***	**K**^**+**^	**α, pore**	**ENSRNOG00000012711**
30	*Kcnq2*	K^+^	α, pore	ENSRNOG00000011624
31	*Kcnq3*	K^+^	α, pore	ENSRNOG00000005206
32	*Kcnq5*	K^+^	α, pore	ENSRNOG00000013781
33	*Kcns1*	K^+^	α, pore	ENSRNOG00000013681
34	*Kcns2*	K^+^	α, pore	ENSRNOG00000011369
35	*Kcns3*	K^+^	α, pore	ENSRNOG00000004899
36	*Kcnv1*	K^+^	α, pore	ENSRNOG00000004117
37	*Kcnab2*	K^+^	β, auxiliary	ENSRNOG00000011550
38	*Kcnab3*	K^+^	β, auxiliary	ENSRNOG00000008480
39	*Kcnip1*	K^+^	β, auxiliary	ENSRNOG00000005365
40	*Kcnip2*	K^+^	β, auxiliary	ENSRNOG00000018018
41	*Kcnip3*	K^+^	β, auxiliary	ENSRNOG00000014152
42	*Kcnj1*	K^+^, inwardly-rectifying (IR)	pore,	ENSRNOG00000008779
43	*Kcnj2*	K^+^, IR	pore,	ENSRNOG00000004720
**44**	***Kcnj3***	**K**^**+**^**, IR**	**pore**	**ENSRNOG00000005369**
45	*Kcnj5*	K^+^, IR	pore	ENSRNOG00000033796
46	*Kcnj9*	K^+^, IR	pore	ENSRNOG00000007645
47	*Kcnj10*	K^+^, IR	pore	ENSRNOG00000007705
48	*Kcnj11*	K^+^, IR	pore	ENSRNOG00000021128
49	*Kcnj12*	K^+^, IR	pore	ENSRNOG00000002303
50	*Scn1a*	Na^+^	α, pore	ENSRNOG00000005989
**51**	***Scn8a***	**Na**^**+**^	**α, pore**	**ENSRNOG00000005309**
52	*Scn1b*	Na^+^	α, pore	ENSRNOG00000021102
53	*Scn2b*	Na^+^	α, pore	ENSRNOG00000016221
54	*Scnb3*	Na^+^	α, pore	ENSRNOG00000006937
55	*Scnb4*	Na^+^	α, pore	ENSRNOG00000026679

The genes that are putative LEF/TCF targets are in bold.

**Figure 2:**
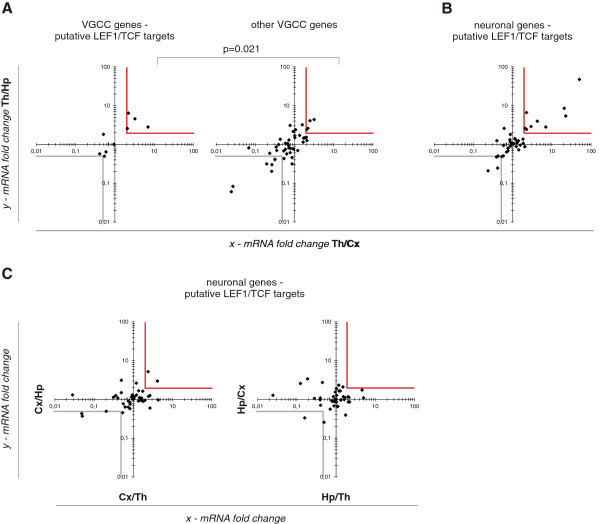
**Gene profiling in the forebrain.** Scatterplots show mean gene expression fold changes between the thalamus, hippocampus, and cortex by RT-qPCR. *p* values (Student’s *t*-test) revealed statistical significance for all fold changes > 2 and < 0.5. A logarithmic scale is used. Red frames surround the plot areas of at least two-fold higher expression in a given brain region compared with the other two regions, gray frames - of at least two-fold lower. (**A**) Expression of VGCC genes in the thalamus *vs.* cortex (*x*-axis) and hippocampus (*y*-axis). (*Left plot*) VGCC genes defined as putative LEF1/TCF targets. (*Right plot*) Remainder of the VGCC genes. The proportions of highly expressed genes in the group of putative LEF1/TCF targets (*left plot*) and in the non-target group (*right plot*) were compared using Fisher’s Exact test, indicating a nonrandom association (*p* = 0.021). (**B**) Expression of all putative neuronal LEF1/TCF targets in the thalamus *vs.* cortex (*x*-axis) and hippocampus (*y*-axis). Notice that many genes are highly expressed in the thalamus. (**C**) Expression of all putative neuronal LEF1/TCF targets. (*Left plot*) Cortex *vs.* thalamus (*x*-axis) and hippocampus (*y*-axis). (*Right plot*) Hippocampus *vs.* thalamus (*x*-axis) and cortex (*y*-axis). Notice that this group of genes is not preferentially expressed in the cortex or hippocampus. *n* = 6 independent biological samples.

The expression of 53 of the 55 VGCC genes was detected in the forebrain; no signal was obtained for *Kcnip1* and *Kcnj1*. Four of nine putative LEF1/TCF target VGCC genes were expressed at least two-fold higher in the thalamus than in the cortex and hippocampus, and none were expressed at a lower level. In the remaining group of 44 genes, four were expressed at a higher level in the thalamus, and six were expressed at a lower level (Figure 2A). Fisher’s Exact test, yielding a *p* value of 0.021, confirmed a correlation between thalamic expression and being a putative LEF1/TCF target. This validated the approach to identifying new LEF1/TCF targets based on combined *in silico* binding site prediction and high expression in the thalamus (i.e., the region with high levels of nuclear β-catenin and LEF1/TCF factors in neurons).

### More than 20% of neuron-specific genes with conserved LEF1/TCF motifs are highly expressed in the thalamus (RT-qPCR arrays)

We next expanded the analysis and profiled the expression of all 41 genes listed as putative neuronal targets of LEF1/TCF (Table 2) in the cortex, hippocampus, and thalamus. Another set of custom-designed RT-qPCR arrays and the previous six independent preparations from each brain region were used. Our aim was to determine which of the predicted targets are highly expressed in the thalamus, suggesting their actual regulation by β-catenin-LEF1/TCF.

**Figure 3:**
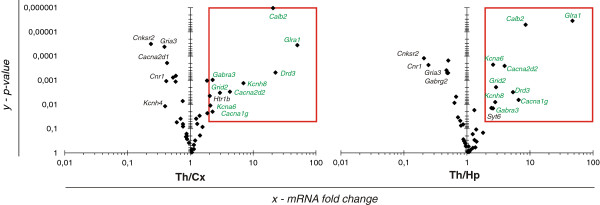
**Expression analysis of putative LEF1/TCF targets in the forebrain.** Volcano plots arrange genes along the dimensions of (*x*) mean expression fold difference between two brain structures and (*y*) *p* value (Student’s *t*-test). A logarithmic scale is used. Red frames surround the plot area, in which the expression in the thalamus is at least two-fold higher than in the other structures, and the difference is statistically significant (*p* < 0.05). The genes inside the frames are considered likely LEF1/TCF targets in the thalamus. On every plot, the genes that met the criterion of a statistically significant two-fold expression difference between the two structures are labeled. Those that are higher in the thalamus *vs.* cortex and hippocampus are in green. *n* = 6 independent biological samples.

The expression of 40 of the 41 genes was detected in the forebrain; no signal was obtained for *Htr3b*. In the thalamus, nine genes (22.5%) were expressed at least two-fold higher than in the cortex and hippocampus (Figure 2B), and the differences were statistically significant (Figure 3). In the cortex *vs.* the other two regions, two genes (5%) were expressed at a higher level, and no single gene was highly expressed in the hippocampus (Figure 2C).

The following genes had higher expression levels in the thalamus: four VGCC genes (*Cacna1g*, *Cacna2d2*, *Kcna6*, and *Kcnh8*, which were also observed in the previous experiment), four genes that represent neurotransmitter receptors (*Drd3*, *Gabra3*, *Glra1*, and *Grid2*), and the *Calb2* gene that encodes the Ca^2+^ buffer calretinin (Figure 3). The genes from two other categories (i.e., the genes that encode synaptic vesicle proteins and structural synaptic proteins; Table 2) did not show specific thalamic expression.

### β-catenin is associated with chromatin at the *Cacna1g, Kcna6, Gabra3, Grid2, and Calb2* loci in the thalamus

**Figure 4:**
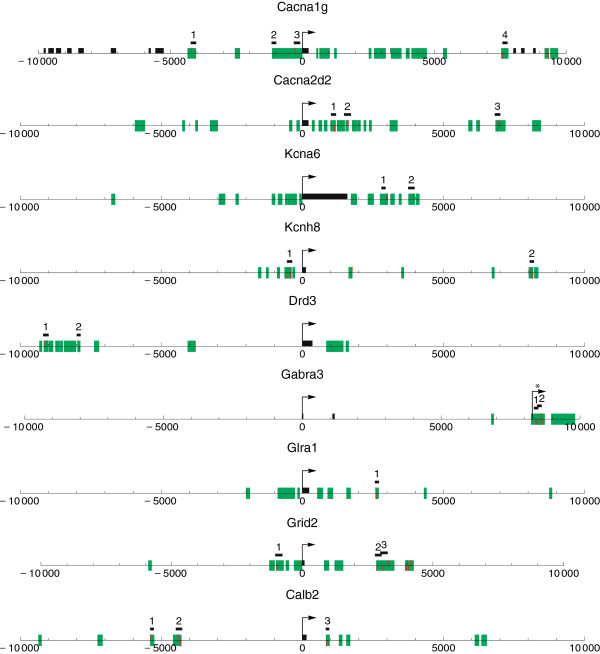
**Positions of LEF1/TCF motifs in the conserved noncoding sequences in the genomic flanks of the transcription start sites of genes selected for experimental validation.** The schemes represent *Drd3*, *Gabra3*, *Glra1*, *Grid2*, *Cacna1g*, *Cacna2d2*, *Kcna6*, *Kcnh8*, and *Calb2* genes. The plots are on the gene strand, nucleotide positions are given relative to the transcription start site (TSS) of each gene, as defined in the Ensemble version used. For *Gabra3*, an alternative TSS prediction, marked with the asterix, based on the NCBI Reference Sequence NM_017069.3, is also shown. CNSs are represented as green rectangles. Positions of LEF1/TCF motifs (analyzed only in the CNSs) are marked as red bars, above or below the axis depending on the strand. Exons within the analyzed flanks (for majority of the genes – only the first exons) are shown as black rectangles on the axis. Amplicons used in the ChiP assay are shown as numbered thick black lines.

Gene profiling in the rat brain allowed us to observe a positive association between the relative expression of the neuronal genes with at least two conserved LEF1/TCF motifs and the presence of β-catenin and LEF1/TCF factors in the brain. To determine whether the β-catenin-LEF1/TCF complex might directly regulate *Cacna1g*, *Cacna2d2*, *Kcna6*, *Kcnh8*, *Drd3*, *Gabra3*, *Glra1*, *Grid2*, and *Calb2*, we analyzed the *in vivo* binding of β-catenin to LEF1/TCF motifs within the CNSs using a chromatin immunoprecipitation (ChIP) assay with designed primers (Figure 4 and Table 4). We also examined the chromatin conformational state of the fragments that contained conserved LEF1/TCF motifs by precipitating them with an antibody specific for acetyl-histone H3 (H3Ac; a hallmark of open chromatin [42,43]). In each ChIP assay, we compared four independent samples of chromatin isolated from the cortex, hippocampus, and thalamus.

**Table 4:** List of primers used in chromatin immunoprecipitation assay

***PCR amplicon***	***Forward primer***	***Reverse primer***
Cacna1g - 1	TCAACCTTGCCAGCAAGTCAAAGC	ACAGGCAAGAGGGCTACATGAACT
Cacna1g - 2	AAAGATGTCAGAGGCCGGTTGG	TCCAGACCGAACCCACTTTCCTAT
Cacna1g - 3	AAGCGAAGAAGCCGGAACAAAGTG	GCTCTAGAGAGCTTGCTGAGTCCC
Cacna1g - 4	GTGCGAAAGTGCGTGGTAAACTGT	AGGCTCCGTGAGTTTGCTGTGAAT
Cacna2d2 - 1	ACCATGCCTTAGCCATATGCCAGT	TCTCCTCAAAGCCGAGGACAGAAA
Cacna2d2 - 2	GGGTTAGTGGGTTGTGGTATTGTC	TTCGAGGAGCTGGGAAGCTAAGAA
Cacna2d2 - 3	TATCTCTGCAGCCAGAATGTGCTC	AGGCTTTGTTTCTCACTGCCAACG
Kcna6 - 1	AAGGTAACTCGGTGGTTCCCACTT	AATGGCAATGTTATGGGAGGGAGG
Kcna6 - 2	TGTCACCCTCATCACTATCCCTGT	AGCTGGGATATGTAGTCTGTTCCC
Kcnh8 - 1	TTACCTGCAGTGACCTGTCTCCT	AAACGCGAGTCAGAGCAACTCC
Kcnh8 - 2	CTAAATGTATTCACTGTTCAAAGC	GTCAGCAAATTCTAAGCATCA
Drd3 - 1	GGCAATTAAACTGAGGATGGTGAG	TCCCTCTGAGGCTCTATCTGCTTT
Drd3 - 2	GCAACAGCAAAGTGGAACTCAG	GTAGCATCAGTCAGTAAGCAAAGGG
Gabra3 - 1	ATTTGGAGCCTGGGTTAGAAGGCA	TACCAAGCCGCTGTGAGATGCTTA
Gabra3 - 2	TAAGCATCTCACAGCGGCTTGGTA	TCTAGAGAAGCTCAGAGAGAAGGG
Glra1 - 1	ACCTTGAAACCAAAGGGAGCATGG	GGGCAAGGAACTGTTGGG
Grid2 - 1	TTTGAGTCCTGCCTTTCTCAGTCC	TAAGAGAAGCGAGTCCGCAAGAGA
Grid2 - 2	TCCTTGCCCTGGTGAAAGAAAGGT	CCTACAGCATGCAGATCGTTAGGA
Grid2 - 3	AGGCTATTCAGAAGTCGCGCTGAT	TGTGCAGTTGACTGGAAATGCCAC
Calb2 - 1	AGACCTAATATGCTTCAGGCTCAG	AGGTGGGATAGAGGGACTGAAAGA
Calb2 - 2	TTCATCCTCCTTTCTGGGAGGCAA	TCCTCAGAGCACCTGCTGTCATTT
Calb2 - 3	TGCTCTACATGGTCTTCACAATGA	GGTCTGGTAGCCACCTCCAA
Gapdh – P	CCGACCTTCACCATCTTGTCT	CTGGCCACGCTAATCTGA
Gapdh - E	TTGTGACAAAGTGGACATTG	AACTTGCCGTGGGTAGAGTC

The positions of the primers are shown in Figure 4.

**Figure 5:**
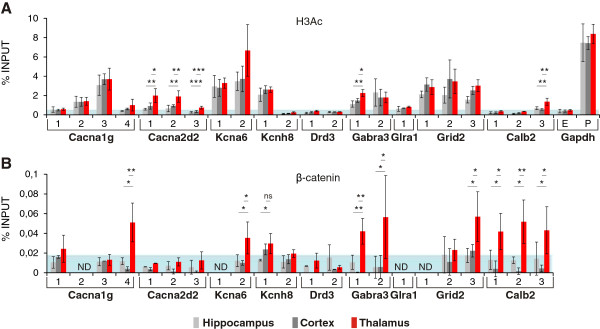
**ChIP analysis of histone acetylation and β-catenin binding to LEF1/TCF motifs of the candidate target genes.** (**A**) The graph shows the mean percentage of input chromatin precipitated with an anti-H3Ac antibody. Fragments of the *Gapdh* promoter (Gapdh-P) and exon (Gapdh-E) were used to determine the signal levels in the case of open and closed chromatin, respectively. The blue area indicates the level of signal for closed chromatin, assessed based on the precipitation of the exonic fragment of *Gapdh*. (**B**) Mean percentage of input chromatin precipitated with an anti-β-catenin antibody. The blue area indicates the level of background, determined with normal IgG. In some cases β-catenin binding to chromatin was not detected (ND). *n* = 4 independent biological samples. Error bars indicate SD. ***p < 0.001, **p < 0.01, *p < 0.05 (ANOVA).

We first assessed the acetylation status of histone H3 at the *Gapdh* promoter and *Gapdh* exon, an open and close chromatin region, respectively. We found high levels of H3Ac in the promoter while much lower levels in the first exon. This showed that our ChIP assays to monitor H3Ac was specific (Figure 5A). We then analyzed the chromatin conformation of our genes of interest in fragments with conserved LEF1/TCF motifs. The chromatin fragments that were in close proximity to TSSs, Cacna1g-3 and Kcnh8-1, appeared to be in an open state. The same was observed for some fragments located distally from the TSSs (e.g., Grid2-1, -2, and −3), whereas other fragments precipitated with low efficiency (< 1%; e.g., Drd-1 and −2), indicating the closed conformation of chromatin (Figure 5A). In most cases, no differences were found between the analyzed brain structures. However, some fragments (e.g., Gabra3-1, Cacna2d2-1, Cacna2d2-2, Cacna2d2-3, and Calb2-3) precipitated significantly more efficiently from the thalamic samples than from the cortex and hippocampus.

We then performed a ChIP assay with an anti-β-catenin antibody. To determine the background, normal immunoglobulin G (IgG) was used, which precipitated < 0.02% of the input. The signals for all of the examined fragments were at background levels in the case of the cortex and hippocampus (Figure 5B). However, when the thalamic samples were used, fragments of *Gabra3*, *Grid2*, *Cacna1g*, *Kcna6*, and *Calb2* precipitated with anti-β-catenin at levels of 0.04-0.1% (Figure 5B), indicating the binding of β-catenin to these fragments. Moreover, for all of these fragments, the differences in chromatin precipitation levels between the thalamic samples and other samples were statistically significant. This indicates that the aforementioned genes can be directly regulated by β-catenin and LEF1/TCF factors. Interestingly, no correlation was observed between the β-catenin-chromatin interaction (Figure 5B) and acetylation status of histone H3 in the chromatin fragments (Figure 5A).

### Attenuation of β-catenin signaling leads to decreases in *Cacna1g, Cacna2d2, Kcna6, Kcnh8, Gabra3, and Calb2* expression in cultured thalamic neurons

Finally, we examined the effect of nuclear β-catenin removal in primary thalamic cultures on the expression of the nine genes identified by gene profiling. The cultures contained both neurons and glia (approximately 1:1), which is vital for the survival of thalamic neurons [38].

**Figure 6:**
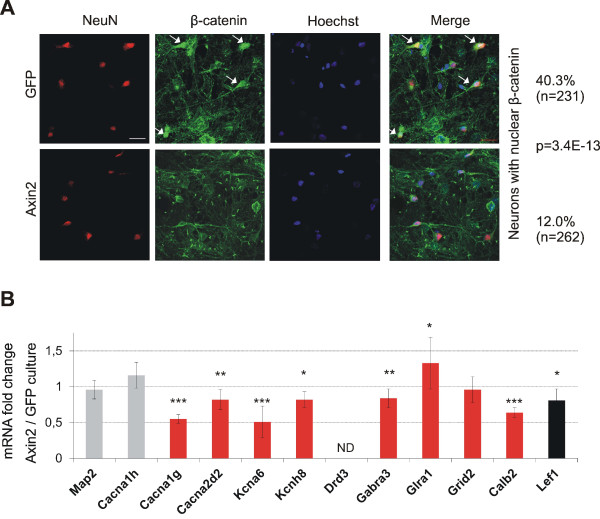
**Expression analysis of the candidate target genes in thalamic neurons (loss-of-function experiment).** (**A**) Subcellular localization of β-catenin in thalamic neurons *in vitro* in control (*Gfp*-expressing; *upper panel*) and *Axin2*-expressing (*lower panel*) cultures. Neuronal marker NeuN is stained red. β-catenin is green, and nuclei are blue. The arrows point to nuclear β-catenin-positive neurons. Scale bar = 20 μm. The percentage of β-catenin-positive neurons in every culture is indicated, with *p* values of the differences (Fisher’s Exact test). (**B**) Expression of the candidate LEF1/TCF1 targets *Cacna1g*, *Cacna2d2*, *Kcna6*, *Kcnh8*, *Drd3*, *Gabra3*, *Glra1*, *Grid2*, and *Calb2*, neuronal marker *Map2*, negative control *Cacna1h*, and positive control *Lef1* in thalamic cultures transduced with *Axin2*-expressing adenoviral vector compared with control (*Gfp*-expressing cultures). The expression levels are relative to the level of *Gapdh*. The graph shows the means of all of the results relative to the control, set at 1. *Drd3* mRNA was not detected (ND). *n* = 9 independent biological samples. Error bars indicate SD. ***p < 0.001, **p < 0.01, *p < 0.05 (Student’s *t*-test).

Thalamic neurons cultured *in vitro* maintain the nuclear localization of β-catenin [38]. To decrease its level, the cultures were treated with an adenovirus that carried *Axin2*, the product of which is a component of the β-catenin destruction complex and as such should reduce its cytoplasmic and nuclear pool. The control cultures were transduced with *Gfp*-expressing adenovirus. The percentage of β-catenin-positive neurons decreased from ~40% to 10% in cultures with ectopic *Axin2* expression (Figure 6A). No nuclear β-catenin was detected in glia.

Afterward, the expression levels of the nine genes and control genes, *Map2*, *Cacna1h*, and *Lef1*, were measured by RT-qPCR. Similar expression levels of the neuronal marker *Map2* in *Gfp*- and *Axin2*- expressing cultures verified the equal survival and condition of neurons (Figure 6B). The level of *Lef1*, serving as a positive control as a classical target of β-catenin, decreased by ~20%, confirming the impairment of β-catenin-dependent transcription in the treated cultures. The expression level of *Cacna1h*, a paralog of *Cacna1g* that lacks conserved LEF1/TCF motifs, was not modified. Of the nine examined genes, we observed a statistically significant decrease in the expression of six genes, *Cacna1g*, *Cacna2d2*, *Kcna6*, *Kcnh8*, *Gabra3*, and *Calb2*, suggesting that these genes are authentic targets of β-catenin. The level of *Glra1* expression increased, and *Grid2* expression did not change. *Drd3* transcription was not detected (Figure 6B).

The actual decreases of the expression levels in given neurons were supposedly several times larger, considering that the changes in the level of nuclear β-catenin occurred in ~30% of the cultured neurons. This percentage was estimated based on the following observations: (*i*) 40% of the neurons were β-catenin-positive in the control, and (*ii*) 12% of the neurons still maintained the nuclear localization of β-catenin after Axin2 adenovirus treatment (Figure 6A). The most pronounced effects were observed for *Cacna1g*, *Kcna6*, and *Calb2*; together with the ChIP results (Figure 5B), this indicates the high importance of β-catenin in the regulation of the expression of these genes in thalamic neurons. The ChIP and expression results were also consistent for *Gabra3* and corroborated the regulation of this gene by β-catenin.

## Discussion

The present study identified five new β-catenin target genes in thalamic neurons, in addition to previously described *Cacna1g*[16]. Three of them, *Kcna6*, *Calb2*, and *Gabra3*, were validated by ChIP *in vivo* and a loss-of-function experiment in cultured neurons, confirming that they might be directly regulated by β-catenin. Two other genes, *Cacna2d2* and *Kcnh8*, also displayed β-catenin-dependence in the latter experiment, although the binding of β-catenin to their regulatory elements was not found. Based on these data, we propose that β-catenin is a regulator of the electrophysiological properties of thalamic neurons in the adult brain.

Numerous genes that we selected *in silico* as potentially regulated by β-catenin belong to expected functional categories: transcription regulation, cell proliferation, morphogenesis, motility, adhesion, differentiation, and programmed cell death. Similar clusters were observed by others in the genes bound by TCF7L2 in a human colorectal cancer cell line [40]. These results support the well-established role of Wnt/β-catenin in development. Interestingly, the genes involved in neuronal differentiation exhibited the highest enrichment scores in our list. This was consistent with a study that identified β-catenin-LEF1/TCF targets based on a ChIP assay in NIH3T3 cells. Thirty percent of the target genes were implicated in developmental processes, and more than half of the targets from this group were involved in neuronal development [44]. Indeed, Wnt signaling has been particularly implicated in central nervous system development, from early brain patterning to embryonic and adult neurogenesis [30-33,45-50].

Although our *in silico* analysis corroborated the involvement of LEF1/TCFs in the regulation of well-known groups of genes, it also identified a group that has not been previously proposed to be a β-catenin-LEF1/TCF target. These were the genes of proteins involved in signal transmission in neurons, including voltage-gated ion channels, neurotransmitter receptors, synaptic vesicle proteins, and synaptic structural proteins. Moreover, we provided experimental evidence of the authentic regulation of some of these genes by β-catenin. The above gene clusters have not yet been identified, probably because the screenings for β-catenin target genes were performed on established cell lines or cancer cells [40,44,51-54]. Additionally, studies of hippocampal neurons, in which β-catenin nuclear translocation was observed after NMDA stimulation, did not attempt to identify specific neuronal targets [35-37].

While examining the β-catenin-chromatin association and acetylation of histone H3, we did not observe any relationship between these two phenomena in the analyzed regions with the LEF1/TCF motif. This suggests that the interaction between the β-catenin-LEF1/TCF complex and DNA might not require the open conformation of chromatin. These results may also suggest that the β-catenin complex does not always increase histone acetylation, although it potentially has such an ability [55,56]. This is consistent with a recent study performed on embryonic stem cells, in which knockdown of *Tcf7* and *Tcf7l2* did not affect the active chromatin conformation of their targets [57]. We also noticed an interesting pattern of LEF1/TCF motif occurrence in the examined genes. The motifs were usually clustered downstream of the first exon and not in the promoter regions. This suggests that the transcription of these genes may be regulated by LEF1/TCF factors by gene looping, which has been demonstrated for the *COX2* and *MMP13* genes, in which LEF1/TCF binding sites were located in the 3′ untranslated region [58].

The neuronal genes with conserved LEF1/TCF motifs that were highly expressed in the thalamus, the regulation of which by β-catenin was confirmed experimentally, encode proteins involved in neuronal excitability. Cav3.1 (encoded by *Cacna1g*), Cavα_2_δ2 (*Cacna2d2*), Kv1.6 (*Kcna6*), Kv12.1/ELK1 (*Kcnh8*), and GABA_A_ receptor 3 (*Gabra3*) are all voltage- or ligand-gated ion channels [59-61]. As such, they underlie the cell membrane conductance of Ca^2+^, K^+^, and Cl^-^ (in the case of the GABA receptor) ions and directly propagate, inhibit or modify electric signals [62-64]. Calretinin, in turn, is an intracellular Ca^2+^-binding protein [65,66] with diverse functions, including the modulation of intrinsic neuronal excitability [67]. We propose that β-catenin contributes to the proper excitability of thalamic neurons by regulating the expression of the above genes. However, more research is required to determine the real impact of β-catenin and LEF1/TCF factors on the expression of the identified genes and electrophysiology of the thalamus.

The other classes of putative neuronal targets of LEF1/TCF (i.e., the genes that encode structural synaptic proteins, mainly with the PDZ domain, and synaptic vesicle proteins) did not show high expression in the thalamus. However, they still might be regulated by β-catenin and LEF1/TCF factors in some subtypes of neurons or under specific physiological conditions because the regulation of gene expression by β-catenin is very much context-dependent [8,44,68,69]. Particularly interesting would be the exploration of this possibility in future research because a membranous fraction of β-catenin interacts with PDZ proteins in synapses and is implicated in synaptic vesicle localization [70-73]. The role of nuclear β-catenin in the regulation of PDZ and synaptic vesicle protein expression might complement the function of membranous β-catenin in neurons.

We do not yet know whether variations in the nuclear level of β-catenin affect the expression of genes that encode VGCCs and neurotransmitter receptors and shape neuronal excitability *in vivo*. If so, then we could speculate that the inappropriate activity of β-catenin might affect proper signal transmission in thalamocortical circuits. Thalamocortical desynchronization underlies absence epilepsy [74], and many anticonvulsant drugs target voltage-gated channels (e.g., T-type Ca^2+^ channels [63]). Specifically, the T-type voltage-gated channel Cav3.1 has been proposed to be implicated in absence seizures [75,76], in addition to the Cavα_2_δ2 regulatory subunit of voltage-gated channels [77] and GABA_A_ receptor 3 [78]. Schizophrenia has also been associated with thalamic dysfunction [79-84]. Moreover, some variants of *Tcf7l2* have been recently shown to be a risk factor in schizophrenia [23,85], and a group of synaptic genes involved in excitability has been found to be associated with the risk of schizophrenia [86]. Interestingly, *Gabra3*-deficient mice display impairments in sensorimotor gating, which is a feature of this disorder [87]. These results suggest a possible role for β-catenin-dependent gene expression in thalamic pathologies, but further *in vivo* studies are required to elucidate this issue.

## Conclusions

We identified a novel group of genes regulated by β-catenin-LEF1/TCF that encode proteins that underlie the transmission of nerve impulses. These results point to a novel role for β-catenin in the thalamus, in which β-catenin is constantly present in the cell nuclei. The identified and confirmed genes are directly involved in neuronal excitability, suggesting that β-catenin and LEF1/TCF transcription factors maintain the proper activity of thalamocortical circuits. The presented results may implicate the involvement of β-catenin transcriptional activity in thalamic pathologies, such as absence epilepsy and schizophrenia.

## Methods

### In silico screening and validation of the method

For each human-rat orthologous gene pair in Ensembl (version 39), 20 kb flanks of the genomic sequence from −10 kb to +10 kb from TSSs in either species were aligned using the AVID global alignment algorithm. Conserved noncoding sequences, defined as sequence windows at least 100 bp long with at least 75% identity between human and rat, were selected as putative regulatory regions. The binding sites for LEF1/TCF were separately predicted using the position weight matrices in the V$LEFF family (Genomatix Matrix Family Library, version 6.2) with the MatInspector program for the human and rat sequences of each CNS pair. V$LEFF motifs with a nonzero number of instances in both the human and rat sequences of the same CNS pair (not necessarily the same number or in the same AVID-aligned position) were considered conserved. To validate the method, the group of genes with at least one or at least two conserved V$LEFF motifs were crossed with known Wnt target genes listed at http://www.stanford.edu/group/nusselab/cgi-bin/wnt/target_genes (accessed January 11, 2011). The Wnt targets that had no CNSs, were indirect targets, or had confusing names were excluded from this analysis.

### GO analysis

The functional annotation tools of DAVID Bioinformatics Resources (http://david.abcc.ncifcrf.gov; accessed January 11, 2011) were used to annotate the genes [88-90]. Gene enrichment in annotation terms was measured by determining the EASE score [91], which is a conservative correction of Fisher’s Exact *p* value, for the proportions of genes that fell into each GO term.

### Animal care

To perform this study, we used Wistar rats: 18 adult males and 12 pregnant females with 19-day-old embryos. Animal care was in accordance with the European Communities Council Directive (86/609/EEC). The experimental procedures were approved by the Local Commission for the Ethics of Animal Experimentation no. 1 in Warsaw (approval no. 883/2008).

### Chromatin isolation, shearing, and immunoprecipitation

Three-month-old male Wistar rats were sacrificed by cervical dislocation, and the brains were removed and sectioned. Approximately 200 mg of cortical, hippocampal, and thalamic tissues obtained from two rats were chopped with a razor blade and fixed at room temperature for 30 min in 1 ml of fresh 3% formaldehyde in phosphate-buffered saline (PBS) supplemented with protease inhibitor cocktail (Roche) in an Eppendorf tube. Fixation was stopped by adding glycerol to a final concentration of 0.125 mM and incubation for a further 10 min to quench formaldehyde. The tissue was then spun down for 5 min at 1,000 × *g* and briefly homogenized in ice-cold PBS supplemented with protease inhibitor cocktail using an Eppendorf-fitting pestle homogenizer. After two cycles of washing in PBS (i.e., homogenization and centrifugation), the samples were lysed in 2.5 ml of lysis buffer (1% sodium dodecyl sulfate, 10 mM ethylenediamine tetraacetic acid [EDTA], 50 mM TrisHCl, pH 8.1, and protease inhibitor cocktail) for 30 min at 4°C and then disrupted in a Potter-Elvehjem homogenizer (~100 strokes). The homogenates were centrifuged for 10 min at 2,400 × *g* to pellet the nuclei, which were then frozen at −80°. After thawing, the samples were diluted in digestion buffer from the ChIP-IT Express Enzymatic kit (Active Motif) and sonicated on ice for 10 × 20 s with 40 s breaks at a 50% duty cycle and 50% power using a Sonopuls Bandeline sonicator to disrupt the nuclei and preshear the chromatin. An enzymatic shearing protocol using the ChIP-IT Express Enzymatic kit was then followed. Shearing was conducted with 40 μl of Enzymatic Shearing Cocktail per one sample for 15 min at 37°C, and the reaction was stopped with EDTA. Finally, the sheared chromatin was separated from debris by centrifugation at 18,000 × *g*. This treatment yielded 125–500 bp DNA fragments. A subsequent immunoprecipitation procedure was performed on an equivalent 30 mg of tissue with 10 μg of anti-rabbit IgG (Sigma-Aldrich), 5 μg of anti-acetyl-histone H3 (rabbit polyclonal; Millipore), or 40 μg of anti-β-catenin (rabbit polyclonal; Santa Cruz Biotechnology) antibodies, according to the Millipore protocol using salmon sperm DNA protein A-agarose (Millipore). Immunoprecipitated DNA was used as a template for RT-qPCR with SYBR Green chemistry. The obtained data were analyzed using the relative quantification method with the 2^-ΔCT^ formula (DCT = CT_input DNA_ - CT_immunoprecipitated DNA_; CT is the cycle threshold) and are presented as a percentage of the input. The primers used in the ChIP assay are listed in Table 4.

### Primary neuronal cultures

Dissociated primary thalamic cells were obtained from the brains of embryonic day 19 Wistar rat embryos as described previously [38]. The cells were plated on coverslips coated with poly-D-lysine (30 μg/ml; Sigma) at a density of 2.5 × 10^5^ cells per well of a 24-well plate in Minimal Essential Medium (MEM; Gibco) supplemented with 10% fetal bovine serum and 0.2 mM glutamine (Sigma). The next day, the medium was replaced with Neurobasal (Gibco) supplemented with B27 (Gibco), 0.5 mM glutamine, 12.5 mM glutamate, and penicillin/streptomycin (Sigma) mixed in a 1:1 ratio with the cortical neuron conditioned medium.

### Adenoviral constructs and neuron transduction

The construction of recombinant adenoviral plasmids that expressed Gfp and Axin2 and the preparation and purification of the adenoviruses were described previously [38]. On day 4 *in vitro*, purified virus suspensions at a multiplicity of infection of 100 were added to each well of a 24-well plate for 6 h at 37°C. The virus solution was then removed and replaced with fresh medium. Seventy-two hours later, the transduced cultures were collected for RNA isolation or fixation.

### Immunofluorescence and microscopic analysis

The immunofluorescence analysis of β-catenin localization was performed as described previously [38]. Briefly, the cells were incubated overnight at 4°C with anti-β-catenin rabbit antibody (1:250; Santa Cruz Biotechnology) in a humid chamber followed by incubation with anti-NeuN mouse antibody (1:150; Chemicon) for 1.5 h at room temperature. All of the antibodies were diluted in 2% bovine serum albumin. The slides were acquired under a confocal microscope (Zeiss LSM5 Exciter). The fluorescence intensity of intracellular β-catenin was analyzed using Cell Profiler Software. Based on a fluorescent histogram under the control conditions, two separate cell populations with low and high intensity were identified, and the intensity threshold between them was established. In an experimental variant, the cells with fluorescence greater than the threshold were counted as β-catenin-positive cells.

### RNA isolation from brain tissue and neuronal cultures

To isolate RNA from fresh brains, the brains from male, 2-month-old Wistar rats were sectioned, and different structures were homogenized separately using a Potter-Elvehjem homogenizer. RNA was extracted with the RNeasy Lipid Tissue Mini kit with additional DNase treatment (Qiagen). For neuronal cultures, the cells from one to three wells of a 24-well plate were pooled together and homogenized with Qiashredders (Qiagen), and RNA was isolated with the RNeasy Plus Mini kit (Qiagen). cDNA was synthesized by reverse transcription (SuperScript III RnaseH-, Invitrogen).

### Gene expression analysis by RT-qPCR

All RT-qPCR reactions were performed using the 7900HT system (Applied Biosystems). Gene profiling in the brain was performed with custom-designed TaqMan Low Density Arrays (Applied Biosystems), hereinafter referred to as RT-qPCR arrays. Approximately 1.5 μg of cDNA was loaded per array (384 assays). The obtained data were analyzed using the relative quantification method with the 2^-ΔCT^ formula (ΔCT = CT_target_ - CT_*Gapdh*_; CT is the cycle threshold). In thalamic cultures, gene expression levels were examined in individual qRT-PCR reactions. For *Cacna1g*, *Cacna2d2*, *Kcna6*, *Knch8*, *Drd3*, *Glra1*, *Gabra3*, *Grid2*, and *Calb1*, commercial primers were used (Qiagen). For *Map2*, commercial primers and FAM dye-labeled TAqMan probes (Applied Biosystems) were used. *Lef1* and *Gapdh* expression was measured with the following primers: *Lef1* (forward, CCCACACGGACAGCGACCTA; reverse, TAGGCTCCTFTTCCTTTCTCT), *Gapdh* (forward, TGACTCTACCCACGGCAAGTTCAA; reverse, ACGACATACTCAGCACCAGCATCA). SYBR Green chemistry was used, with the exception of *Map2*, for which TaqMan was used (Applied Biosystems). The obtained data were quantified using the relative standard method.

### Statistical analysis

Three types of statistical tests were used. Two-tailed Fisher’s Exact test was used to verify the enrichment of genes with LEF1/TCF binding sites with the known Wnt targets (Figure 1). This test was also performed to calculate *p* values for the relationships between high expression in the thalamus and being a putative LEF1/TCF target (Figure 2; *n* = 6), and compare the percentage of β-catenin-positive neurons in two types of thalamic cultures (Figure 6A).

To calculate *p* values for differences in gene expression levels between two brain regions, we ran two-tailed paired Student’s *t*-test, using ΔCT values for the results obtained with the RT-qPCR arrays (Figure 3; *n* = 6) or using relative values for the results obtained with individual RT-qPCRs (Figure 6; *n* = 9).

To calculate *p* values in the ChIP experiment, in which we compared three brain regions at the same time, we used repeated measures ANOVA followed by Tukey’s *post hoc* test (Figure 5; *n* = 4).

## Abbreviations

CNS: Conserved noncoding sequence; GABA: The γ-aminobutyric acid; GO: Gene ontology; RT-qPCR: Quantitative real-time polymerase chain reaction; TSS: Transcription start site; VGCC: Voltage-gated cation channel.

## Competing interests

The authors declare no conflicts of interest.

## Authors’ contributions

MBW conceived, designed, and coordinated the study, drafted the manuscript, and performed the GO analysis, ChIP assays, and gene expression analysis. AN designed the VGCC arrays, produced adenoviral vectors, and performed viral transduction. MD performed *in silico* screening. KM performed immunofluorescence and microscopic analysis. AN and KM grew the primary cultures. JK discussed and reviewed the project during its implementation and participated in drafting the manuscript. All of the authors critically read and approved the manuscript.

## Supplementary Material

Additional file 1 Excel file with a list of 428 rat genes with at least two LEF1/TCF motifs in the same CNS. Click here for file
